# Dyspnea after pulmonary embolism: a nation-wide population-based case–control study

**DOI:** 10.1177/20458940211046831

**Published:** 2021-09-30

**Authors:** Lars T. Nilsson, Therese Andersson, Flemming Larsen, Irene M. Lang, Per Liv, Stefan Söderberg

**Affiliations:** 1 Department of Public Health and Clinical Medicine, Unit of Medicine, Umeå University, Umeå, Sweden; 2 Department of Molecular Medicine and Surgery, Section of Clinical Physiology, Karolinska Institute and Department of Clinical Physiology, Karolinska University Hospital, Stockholm, Sweden; 3 Department of Internal Medicine II, Division of Cardiology, Vienna General Hospital, Medical University of Vienna, Vienna, Austria; 4 Department of Public Health and Clinical Medicine, Section of Sustainable Health, Umeå University, Umeå, Sweden

**Keywords:** dyspnea, pulmonary embolism, pulmonary hypertension, chronic thromboembolic pulmonary hypertension (CTEPH), post-pulmonary embolism syndrome

## Abstract

Dyspnea is common after a pulmonary embolism. Often, but not always, the dyspnea can be explained by pre-existing comorbidities, and only rarely by chronic thromboembolic pulmonary hypertension (CTEPH). CTEPH is probably the extreme manifestation of a far more common condition, called the post-pulmonary embolism syndrome. The purpose of this retrospective study was to investigate the prevalence and predictors of dyspnea among Swedish patients that survived a pulmonary embolism, compared to the general population. All Swedish patients diagnosed with an acute pulmonary embolism in 2005 (n = 5793) were identified via the Swedish National Patient Registry. Patients that lived until 2007 (n = 3510) were invited to participate. Of these, 2105 patients responded to a questionnaire about dyspnea and comorbidities. Data from the general population (n = 1905) were acquired from the multinational MONItoring of trends and determinants in CArdiovascular disease health survey, conducted in 2004. Patients with pulmonary embolism had substantially higher prevalences of both exertional dyspnea (53.0% vs. 17.3%, odds ratio (OR): 5.40, 95% confidence intervals (CI): 4.61–6.32) and wake-up dyspnea (12.0% vs. 1.7%, OR: 7.7, 95% CI: 5.28–11.23) compared to control subjects. These differences remained after adjustments and were most pronounced among younger patients. The increased risk for exertional dyspnea and wake-up dyspnea remained after propensity score matching (OR (95% CI): 4.11 (3.14–5.38) and 3.44 (1.95–6.06), respectively). This population-based, nation-wide study demonstrated that self-reported dyspnea was common among patients with previous pulmonary embolism. This finding suggested that a post-pulmonary embolism syndrome might be present, which merits further investigation.

## Introduction

Pulmonary embolism (PE) is a common disease that is often preceded and followed by considerable comorbidities. Many studies have investigated the short-term consequences after an acute PE, but less attention has been directed to the long-term effects of a PE. We recently published a nation-wide study on all Swedish patients (i.e. unselected patients with a wide range of comorbidities) that were diagnosed with an acute PE in 2005. We found that the four-year mortality of those patients was more than double (49% vs. 22%) that of age- and sex-matched controls.^1^

The most feared complication after the acute PE phase is chronic thromboembolic pulmonary hypertension (CTEPH), a condition associated with severe functional impairment and high mortality.^2,3^ Due to its severity, many studies have investigated the epidemiology of CTEPH after an acute PE. A recently published meta-analysis of CTEPH after an acute PE found that the CTEPH incidence was 0.6% among “all comers”, 3.2% among “survivors”, and 2.8% among “survivors without major comorbidities”.^4^ The Pulmonary Embolism International Thrombolysis (PEITHO) randomized trial compared thrombolysis vs. anticoagulation in patients with intermediate-risk PE. At a three-year follow-up, they found a similar CTEPH incidence (2.7%) between the two treatment groups.^4^

However, Klok et al. proposed that CTEPH should be viewed as the extreme manifestation of a far more common condition, called the post-PE syndrome,^5,6^ which is characterized by permanent changes in pulmonary circulation, gas exchange and/or cardiac function, and is associated with dyspnea and loss of physical capacity. However, in the post-PE syndrome, pulmonary arterial hypertension does not meet the CTEPH criteria at rest.^5^ This has been addressed in the 2019 European Society of Cardiology guidelines which encompasses the follow-up strategy and diagnostic work-up for the entire spectrum of long-term sequelae of acute PE.^7^

Dyspnea and functional limitations are frequent complaints after a PE, despite adequate anticoagulation.^8–11^ In one of the larger long-term follow-up studies that included 607 patients with PE, 36% reported exertional dyspnea at 3.6 years after the PE diagnosis, and of these, 76% reported that dyspnea had either developed or worsened after the acute PE.^10^ Similar findings were reported in the PEITHO trial, where 33% of all patients (n = 709) reported exertional dyspnea at a three-year follow-up, with no significant difference between study groups.^12^ In another study, among 109 previously healthy patients that experienced a first-time PE, 25% exhibited functional limitations six months later (i.e. a six-minute walk distance < 330 m, a New York Heart Association functional classification >2, or both).^11^ In a similar study on 162 patients selected less strictly, the prevalence of functional limitations (with the same definition) was 40–49% at six months after the PE.^13^

Consistent with the post-PE syndrome hypothesis, it has been demonstrated that not all PE patients exhibit complete thrombus resolution, despite adequate anticoagulation. In 2006, a systematic review found that more than 50% of patients with PE had residual pulmonary thrombi at six months after diagnosis; moreover, after six months, the resolution of thrombi appeared to reach a plateau phase.^14^ More recent studies have reported a lower prevalence of incomplete thrombus resolution, ranging from 20 to 30%, after six months of anticoagulation treatment.^9,15–18^

Analogous to incomplete thrombus resolution and consistent with the post-PE syndrome hypothesis, long-term echocardiographic studies have shown that the prevalence of persistent right ventricular dysfunction (RVD) after PE exceeded the prevalence of RVD expected with CTEPH alone.^11,13,19^ In the PEITHO trial, 44% of patients exhibited one or more echocardiographic signs of pulmonary hypertension or RVD after a three-year follow-up.^12^

However, the post-PE syndrome has not yet been clinically validated and it remains largely unknown to what extent acute PE episodes are causally related to dyspnea, functional impairments, and changes in pulmonary circulation and cardiac function.

In the present study, we collected data on Swedish patients that survived an acute PE that occurred in 2005. We aimed to determine the prevalence of dyspnea and its determinants in a large, unselected, post-PE population. We hypothesized that, after a PE, patients would report dyspnea more often than controls, and that this dyspnea would not be explained by comorbidities.

## Methods

### Subjects

In Sweden, all in-patient care and concomitant data are registered in the Swedish National Patient Registry (NPR), which is hosted by the Swedish National Board of Health and Welfare. This registry was inaugurated in 1984, and it maintains over 99% national coverage, because reporting is mandatory by law. The validity of the NPR was proven to be high, in general.^20^ The validity of the PE diagnosis was also evaluated specifically, and the positive predictive value was 81%.^21^

We used the Swedish modification of the 10th revision of the International Classification of Diseases (ICD-10-SE) to identify patients admitted to any Swedish hospital with an acute PE as the main or subsidiary diagnosis during 2005 (post-PE group). We also collected data on comorbidities (i.e. cancer, congestive heart failure (CHF), atrial fibrillation (AF), ischemic heart disease (IHD), and cerebrovascular disease (CVD)) that were registered in the NPR in the eight years prior to the index PE.

In 2007, all Swedish hospital units responsible for treating patients with PE in 2005 were asked for permission to contact those patients and for documentation about the original PE. All units approved the study, but some patients were not deemed suitable for follow-up by the treating unit. All the remaining patients that survived, according to the Swedish population registry, were sent an invitation to participate in a follow-up study. The invitation included a questionnaire and a consent form, which were to be returned to the study center. Patients that did not respond to a written re-invitation were contacted by telephone by a study nurse and asked questions for a non-responder analysis.

**Fig. 1.**
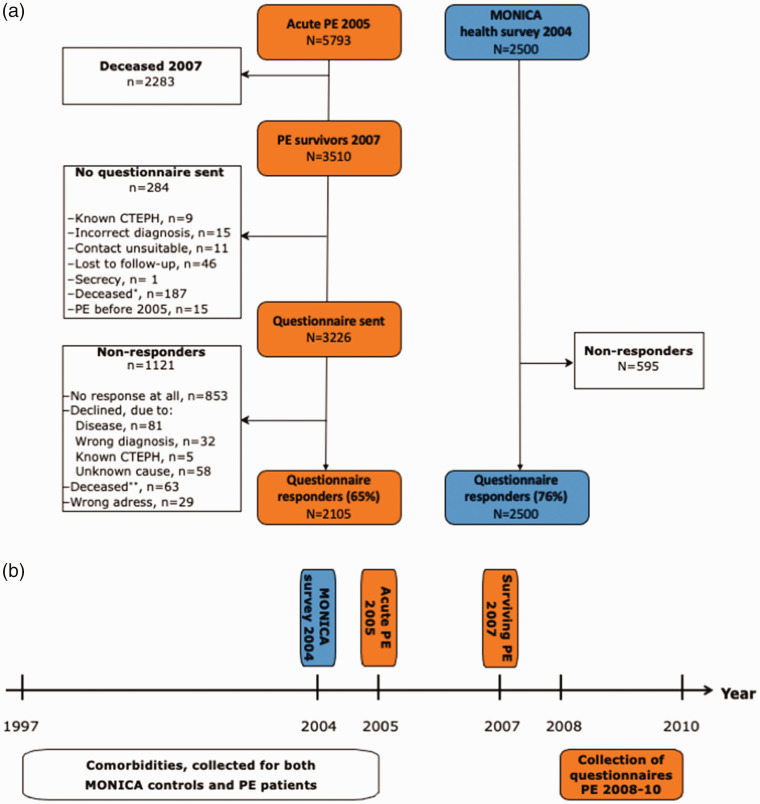
(a) Flow chart illustrates the patient inclusion process. *Deceased between 2007 and sending the questionnaire; **deceased after sending the questionnaire. (b) Timeline for data collection.

For a control group, we recruited participants in the Northern Sweden World Health Organization multinational MONItoring of trends and determinants in CArdiovascular disease (MONICA) health survey, conducted in 2004. Subjects aged 25 to 75 years that lived in the two most northern counties of Sweden (target population aged 25 to 75 years was 312,000) were randomly selected from the population registry. Details of the sampling and selection process were presented previously.^22^ As for patients with PE, we retrieved data on relevant comorbidities from the NPR. The selection process is summarized in Fig. 1a, and timeline for data collection is illustrated in Fig. 1b.

This study complied with the Declaration of Helsinki and was approved by the Regional Ethics Review Board in Umeå, Sweden (07-074). The Ethics Board at the Swedish National Board of Health and Welfare reviewed and approved the extraction of data from the Swedish NPR. Written consent was obtained from all participants.

### Questionnaire

A two-part questionnaire was sent to patients in the post-PE group. The first part was identical to the questionnaire given to the control group in the Northern Sweden MONICA health survey in 2004. This part included questions regarding dyspnea, lifestyle (previous and present smoking), and selected comorbidities (angina pectoris, myocardial infarction, coronary intervention, chronic pulmonary obstructive disease (COPD), hypertension, previous heart valve surgery). The questions related to dyspnea were:

1. “Do you experience shortness of breath when climbing two stairs, or equivalent, at the same pace as people of the same age? Yes/No”
2. “Do you wake up because of shortness of breath? Yes, often/Yes, sometimes/No”.

The second part of the questionnaire was specifically designed for the patients in the post-PE group. These questions focused on health changes after the PE diagnosis, the degree of dyspnea, and the presence of specific comorbidities related to CTEPH development.

### Statistical methods

Categorical variables are expressed as frequencies and percentages. Differences between groups were investigated with Pearson Chi-square tests. Continuous variables were inspected visually for normality on histograms and Q–Q plots, and they were also tested with the Kolmogorov–Smirnov test. Variables with a normal distribution are expressed as the mean ± standard deviation; variables with a non-normal distribution are expressed as the median and interquartile range (IQR). Differences between groups were tested with independent-samples Student t-tests, when appropriate.

Two separate logistic regression models were created, based on a hierarchical method, with results from questions 1 and 2 as dependent variables. Answers to question 2 “Yes, often” and “Yes, sometimes” were clustered as “Yes”, to perform the binary logistic regression. Patients in the post-PE group that were  < 25 or >75 years old were excluded in all comparisons between cohorts and in the regression models, because the control group (from the MONICA survey) only included subjects aged 25–75 years.

All available covariates were first tested with univariable logistic regression. For IHD and coronary interventions, both self-reported data and diagnostic data from the NPR were available, but in the regression models we only used diagnostic data from the NPR. All variables were then tested in forced entry multivariable logistic regression models. We investigated potential interactions between cohort (i.e. the post-PE group and the MONICA control group) and all covariates. Interactions were tested in the multivariable models, only when they were deemed clinically relevant, and they were statistically significant (*p*  < 0.05). Final models were examined for goodness-of-fit (standardized residuals, deviance statistics), multicollinearity, and influence statistics (Cook’s distance, leverage statistics, and DFbeta).

As a confirmatory analysis, propensity score matching was performed, where propensity scores for belonging to the PE group for each individual were estimated using logistic regression. The propensity scores were conditioned on the following covariates: sex, age, COPD, Heart valve surgery, hypertension, present smoking, previous smoking, CHF, cancer, IHD, CVD, and AF. Matching of participants were performed in 1:1 ratio by matching samples with the smallest average absolute distance across all the matched pairs which resulted in 2850 pairs. Conditional logistic regression was used to estimate the odds ratio (OR) of exertional dyspnea and wake-up dyspnea between PE group and control group, adjusting for the propensity score using restricted cubic splines with three nodes at the 10:th, 50:th, and 90:th percentiles. To account for modification of age on the cohort effect, propensity scores were also estimated using the same procedure except for leaving out age as a covariate. Unconditional (non-matched) logistic regression models were thereafter fitted with exertional dyspnea and wake-up dyspnea, respectively, as dependent variables and cohort, age and an interaction with age×cohort interaction as independent variables, while adjusting for the propensity score. OR for the cohorts with 95% confidence intervals (CI) were estimated, conditioning on different values of age.

Data were analyzed with IBM SPSS statistics software version 25 and R version 4.0.3. For the propensity score matching, the function matchit from the R package MatchIt was used.^23^

## Results

We identified a total of 5793 unique patients that experienced a PE during 2005. Of those, 3510 patients remained alive in 2007. After contacting the diagnosing units, 284 patients were excluded by the diagnosing units for reasons listed in Fig. 1a. Finally, 3226 patients were invited to participate. After re-invitations, a total of 2105 patients (65%) responded to the questionnaire. The response rate varied with age, 36 (55%) patients younger than 25 years, 1524 (69%) patients aged between 25 and 75 years, and 545 (44%) patients older than 75 years responded. A summary of the inclusion process is shown in Fig. 1a and b. The median duration between the PE diagnosis and questionnaire completion was 3.4 (IQR 0.7) years. Non-responders were significantly older and had more comorbidities than responders, and the non-responder group included a larger proportion of women than the responder group, see Supplementary Table 1. For the control group (participants in the 2004 MONICA health survey), 2500 subjects were invited and 1905 participated (76%).

**Fig. 2.**
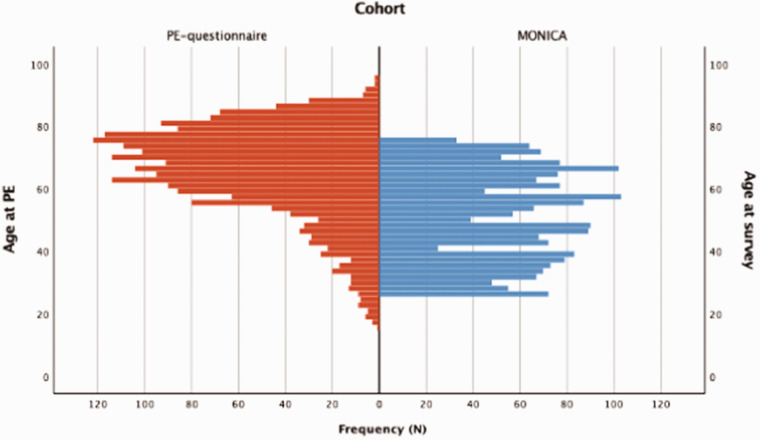
Age distributions of patients in the post-PE group at the time of the survey (n=2105, blue) and for control subjects at the time of the MONICA survey (n=1905, red).

The age distributions differed between the post-PE and control groups, see Fig. 2. The post-PE group were older than the control group, even after excluding patients aged less than 25 years or more than 76 years (mean age±standard deviation: 60.6 ±11.7 vs. 51.0±14.1 years, respectively; *p* < 0.001). Additional subject characteristics are summarized in Table 1. Differences between included and excluded (because of low and high age) post-PE patients are summarized in Supplementary Table 2.

**Table 1.** Characteristics of patients in the post-PE group (after excluding patients aged <25 and >76 years) and in the control group (MONICA).

Characteristic	Post-PEN=1524	MONICAN=1905	*p*
Age at survey, mean (SD)	60.6 (11.7)	51.0 (14.1)	<0.001
Female sex	677 (44.4)	975 (51.2)	<0.001
Self-reported symptoms/diagnoses			
Exertional dyspnea	774 (53.0)	321 (17.3)	<0.001
Wake-up dyspnea	181 (12.0)	33 (1.7)	<0.001
Angina CCS 2	264 (17.8)	103 (5.5)	<0.001
Angina CCS 3–4	117 (8.0)	33 (1.7)	<0.001
Chest pain >30 min	238 (16.1)	177 (9.4)	<0.001
MI, self-reported	133 (9.2)	62 (3.3)	<0.001
Suspected MI, self-reported	46 (3.2)	19 (1.0)	<0.001
PCI/CABG	108 (7.2)	54 (2.8)	<0.001
Heart valve surgery	16 (1.1)	8 (0.4)	0.026
Hypertension	728 (48.5)	536 (28.3)	<0.001
COPD	128 (8.5)	17 (0.9)	<0.001
Present smoking	139 (9.2)	327 (17.3)	<0.001
Previous smoking	754 (50.1)	613 (38.5)	<0.001
Pre-survey diagnoses, according to NPR			
Congestive heart failure	66 (4.3)	8 (0.4)	<0.001
Cancer, any	161 (10.6)	141 (7.4)	0.001
Ischemic heart disease	149 (9.8)	248 (2.5)	<0.001
Cerebrovascular disease	73 (4.8)	19 (1.0)	<0.001
Atrial fibrillation	128 (8.4)	22 (1.2)	<0.001
Pulmonary embolism before 2005	140 (9.2)	5 (0.3)	<0.001

Data shown are numbers (%) of patients, unless otherwise indicated. The denominator for each ratio (%) is the number of valid responses for each item. PE: pulmonary embolism; MONICA: multinational MONItoring of trends and determinants in CArdiovascular disease health survey; CCS: Canadian Cardiovascular Society; MI: myocardial infarction; PCI: percutaneous coronary intervention; CABG: coronary artery bypass graft surgery; COPD: chronic obstructive pulmonary disease; NPR: Swedish National Patient Registry. *p*-Values were based on Students t-test or Pearson χ^2^ test.

The post-PE group had significantly higher prevalences of both dyspnea upon exertion (53.0% vs. 17.3%, *p* < 0.001) and wake-up dyspnea (12.0% vs. 1.7%, *p* < 0.001) than the control population. Results from the univariable logistic regression are summarized in Supplementary Tables 3 and 4. We found no significant interactions between study cohort and the other covariates, except for age and study cohort (i.e. age×cohort interaction in the post-PE vs. MONICA groups). Consequently, the age×cohort interaction was included in the multivariable regression models, which are summarized in Tables 2 and 3.

**Table 2.** Multivariable analysis of associations between comorbidities and the risk of exertional dyspnea.

Comorbidity	*p*	OR	95% CI
Study cohort (post-PE/MONICA)	<0.001	25.19^a^	10.83–58.58
COPD (Yes/No)	<0.001	6.27	3.68–10.67
IHD (Yes/No)	<0.001	2.13	1.47–3.07
CHF (Yes/No)	0.013	2.25	1.19–4.25
AF (Yes/No)	0.052	1.49	1.00–2.22
Hypertension (Yes/No)	<0.001	1.79	1.50–2.15
CVD (Yes/No)	0.231	0.72	0.42–1.24
Cancer (Yes/No)	0.188	0.82	0.61–1.10
Heart valve surgery (Yes/No)	0.619	1.29	0.48–3.49
Present smoking (Yes/No)	0.346	1.19	0.83–1.71
Previous smoking (Yes/No)	0.234	1.12	0.93–1.34
Age (1-year age groups)	<0.001	1.04^a^	1.03–1.05
Women vs. men	<0.001	1.98	1.65–2.37
Study cohort×Age	<0.001	0.97^a^	0.96–0.98

Data shown are odds ratios (OR) with 95% confidence intervals (CI). The odds that exertional dyspnea was related to the indicated comorbidities were tested with multivariable logistic regression analysis, based on both the post-PE and MONICA control groups. PE: pulmonary embolism; MONICA: multinational MONItoring of trends and determinants in CArdiovascular disease health survey; COPD: chronic obstructive pulmonary disease; IHD: ischemic heart disease; CHF: congestive heart failure; AF: atrial fibrillation; CVD: cerebrovascular disease.

^a^A significant interaction was found between the study cohort and age; thus, the related ORs could not be interpreted as average effects.

**Table 3.** Multivariable analysis of association between comorbidities and the risk of wake-up dyspnea.

Comorbidity	*p*	OR	95% CI
Study cohort (post-PE/MONICA)	<0.001	119.94^a^	14.81–971.35
COPD (Yes/No)	<0.001	4.30	2.72–6.79
IHD (Yes/No)	0.002	2.13	1.34–3.39
CHF (Yes/No)	0.289	1.44	0.73–2.83
AF (Yes/No)	0.003	2.15	1.29–3.57
Hypertension (Yes/No)	0.004	1.60	1.16–2.21
CVD (Yes/No)	0.246	1.47	0.77–2.84
Cancer (Yes/No)	0.366	0.78	0.45–1.34
Heart valve surgery (Yes/No)	0.868	1.12	0.30–4.25
Present smoking (Yes/No)	0.129	1.52	0.89–2.63
Previous smoking (Yes/No)	0.058	0.72	0.51–1.01
Age (1-year age groups)	0.003	1.05^a^	1.02–1.08
Women vs. men	0.001	1.69	1.24–2.32
Study cohort×Age	0.001	0.95^a^	0.91–0.98

Data shown are odds ratios (OR) with 95% confidence intervals (CI). The odds that wake-up dyspnea was related to the indicated comorbidities were tested with multivariable logistic regression analysis, based on both the post-PE and MONICA control groups. PE: pulmonary embolism; MONICA: multinational MONItoring of trends and determinants in CArdiovascular disease health survey; COPD: chronic obstructive pulmonary disease; IHD: ischemic heart disease; CHF: congestive heart failure; AF: atrial fibrillation; CVD: cerebrovascular disease.

^a^A significant interaction was found between the study cohort and age; thus, the related ORs could not be interpreted as average effects.

**Fig. 3.**
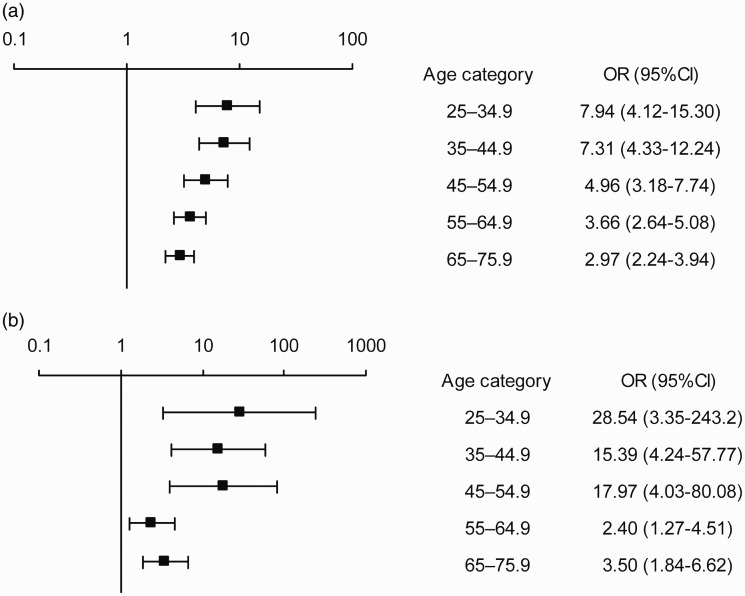
(a) Fully adjusted risk of exertional dyspnea in the post-PE group compared to the MONICA control group, stratified by age. Data are presented as odds ratio (OR) with 95% confidence intervals. (b) Fully adjusted risk of wake-up dyspnea in the post-PE group compared to the control group, stratified by age. Data are presented as odds ratio (OR) with 95% confidence intervals.

Due to the significant interaction between age and cohort, we stratified the risk of dyspnea into 10-year age groups for each cohort. Then, we created separate models for each stratum, and adjusted the models for all significant covariates and interactions. Fig. 3a and b show that, in each age-stratum, dyspnea occurred significantly more often of the post-PE group than in the control group, and the difference between groups was most pronounced among younger subjects. See Supplementary Tables 5 and 6 for more data about numbers in each age-stratum.

**Fig. 4.**
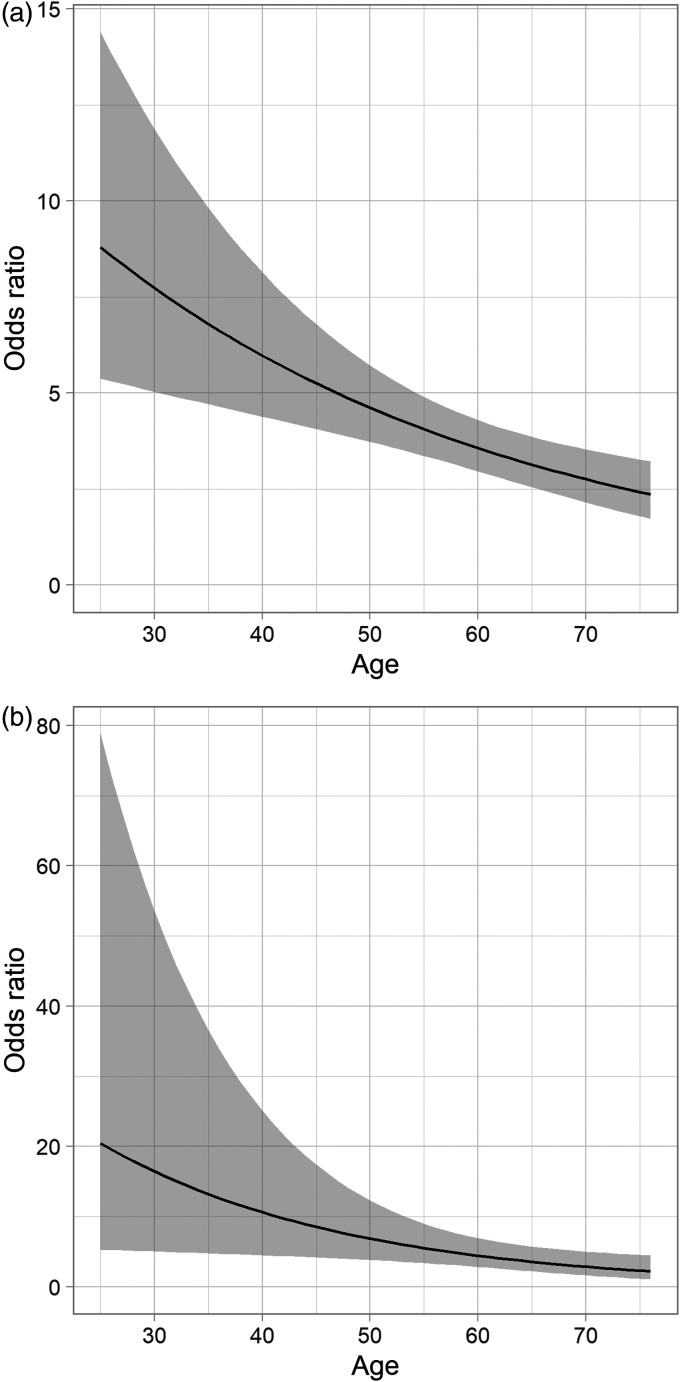
Odds ratio for (a) exertional dyspnea and (b) wake-up dyspnea comparing post-PE group to the MONICA control group, conditioned on age and adjusted for propensity scores. The gray band represents the 95% confidence intervals.

To confirm the findings, we performed a propensity score matching resulting in 2850 matched pairs. The OR for exertional dyspnea belonging to the post-PE group was 4.11 (95% CI: 3.14–5.38). For wake-up dyspnea, the corresponding OR was 3.44 (95% CI: 1.95–6.06). This is further illustrated in Fig. 4a and b showing the propensity score adjusted OR, when including an age interaction to the cohort effect in the logistic regression.

The second part of the questionnaire was answered exclusively by patients in the post-PE group. The degree of exertional dyspnea and changes in health condition at 3.4 years after PE are summarized in Supplementary Table 7. We found that 45% of these patients reported improved health and 22% reported deteriorated health. Moreover, most health changes occurred in the first six months after PE. Women were more dyspneic than men, and more frequently reported deterioration and slow improvements in health. A total of 793 patients in the post-PE group reported no comorbidities. We determined the prevalence of self-reported comorbidities among the remaining 1312 patients. Among these patients, we evaluated the association between each comorbidity and exertional dyspnea, after adjusting for age and sex, see Supplementary Table 8. The most common comorbidities were heart disease (14.5%), cancer (7.9%), CVD (7.5%), and pulmonary disease (6.1%).

## Discussion

This nation-wide, population-based, case–control study is, to date, the largest published study to evaluate the long-term effects of a PE. The main finding of this study was that patients that had experienced a PE had significantly higher prevalences of both exertional dyspnea and wake-up dyspnea compared to controls. The post-PE group also had higher prevalences of self-reported comorbidities and dyspnea-related diagnoses, compared to controls. These comorbidities and diagnoses were however not able to fully explain the differences in dyspnea prevalences. This was illustrated by the significant associations between study cohort and dyspnea in multivariable analyses, i.e. after adjusting for age, sex, smoking habits, and multiple comorbidities.

We observed a 53% prevalence of exertional dyspnea in the post-PE group. This prevalence was somewhat higher than those reported in previous studies, probably due to differences in instruments used to measure dyspnea^10^ and study designs.^12^ One previous study also attempted to assess etiology of post-PE dyspnea and found the dyspnea unlikely to be related to PE and mainly caused by pre-existing comorbidities.^10^ The design of that study did however not allow for a multifactorial explanation of dyspnea: dyspnea could not be classified as PE related if patients had another dyspnea explaining diagnosis. On the contrary, the results of our study suggested that acute PE was indeed an independent risk factor for dyspnea, and that a substantial proportion of patients developed persistent dyspnea related to the acute PE. Probably this proportion covers patients from the entire spectrum of the post-PE syndrome including the 0.6–3.2% that develop CTEPH according to previous studies.^4^ Our findings thus add novel knowledge, since previous studies have either been very small^9^ or have lacked a control group without PE.^8,10^

Furthermore, the highest odds of both exertional dyspnea and wake-up dyspnea were found in the lowest age groups. These results suggested that the association between dyspnea and prior PE was stronger in patients with fewer comorbidities.

As expected, we found that dyspnea was associated with several comorbidities, including chronic obstructive pulmonary disease, IHD, AF, and hypertension. However, somewhat surprisingly, cancer was not associated with either exertional or wake-up dyspnea, and CHF was associated with exertional dyspnea, but not with wake-up dyspnea. These results suggested that different mechanisms might be involved in wake-up dyspnea and exertional dyspnea.

In this study, we found that women were more likely than men to report deteriorations in health, delayed improvement, and dyspnea after PE. Moreover, the recovery from acute PE seemed to be less favorable among women than among men. However, these findings should be interpreted with caution as there were more women among the non-responders and there may have been a bias in the women responding to the questionnaire.

Most changes in health conditions occurred during the first six months after the acute PE, consistent with previous findings.^24^ However, a small proportion of patients reported late health changes that occurred more than six months after the acute PE.

Interestingly, we found that dyspnea was associated with many previously described risk factors for CTEPH,^25^ such as a long-term intravenous line, inflammatory bowel disease, ventricular shunt, and pacemakers. This supports the theory of CTEPH being an extreme manifestation of the post-PE syndrome. In contrast, cancer and splenectomy were not associated with dyspnea. Thus, the risk factors for dyspnea associated with the post-PE syndrome might in part differ from the risk factors for CTEPH.

The strengths of this study were the large-scale approach and its population-based design. The usage of two different statistical approaches showing that a previous PE is significantly associated with self-reported dyspnea strengthens the robustness of our findings, despite that the propensity score matching procedure reduces the number of available subjects, and that the ORs represent an average risk over the whole age-range not taking interaction into account.

This study had also some limitations. Although we considered the PE diagnosis in NPR to be sufficiently valid for a registry study like this, some misclassification may occur. The response rate was high (65% overall, and 69% in the age group 25 to 75 years) in this study, and non-responders were more often women, older and had more comorbidities than responders. We thus expect that the reported prevalences of dyspnea are not overestimated, although selection bias cannot be fully ruled out. Also, we did not assess the baseline dyspnea before PE; thus, we could not investigate whether the reported changes in health conditions reflected PE-related health. Due to the retrospective approach, we had no access to cardiopulmonary exercise testing or other objective measures of dyspnea or functional limitation, echocardiograms or biomarkers in the study population. Moreover, no data were available on diagnostic methods, PE severity or given treatment. The PE population in this study had their PE events in 2005 and it is possible that new treatment guidelines as well as the introduction of direct oral anticoagulants after the study period might influence dyspnea prevalences. Finally, we could not adjust for some diagnoses that could have contributed to dyspnea, including anemia, pulmonary disease other than COPD, pulmonary hypertension, obesity, anxiety, or obstructive sleep apnea.

Our findings supported the post-PE syndrome hypothesis, because we demonstrated an association between acute PE and self-reported dyspnea at a median of 3.4 years after the PE event, independent of concomitant dyspnea-related comorbidities. Future research should aim at validating the clinical relevance of post-PE syndrome by prospective investigation of dyspnea, functional limitation, incomplete thrombus resolution, RVD and their relations to long-term morbidity and mortality in large cohorts.

## Supplemental Material

sj-pdf-1-pul-10.1177_20458940211046831 - Supplemental material for Dyspnea after pulmonary embolism: a nation-wide population-based case–control study Click here for additional data file. Supplemental material, sj-pdf-1-pul-10.1177_20458940211046831 for Dyspnea after pulmonary embolism: a nation-wide population-based case–control study by Lars T. Nilsson, Therese Andersson, Flemming Larsen, Irene M. Lang, Per Liv and Stefan Söderberg in Pulmonary Circulation

## References

[1] Andersson T Söderberg S. Incidence of acute pulmonary embolism, related comorbidities and survival; analysis of a Swedish national cohort. BMC Cardiovasc Disord 2017; 17: 155. 28615009 10.1186/s12872-017-0587-1 PMC5471722

[2] McNeil K Dunning J. Chronic thromboembolic pulmonary hypertension (CTEPH). Heart 2007; 93: 1152–1158. 17699182 10.1136/hrt.2004.053603 PMC1955041

[3] Fedullo P Kerr KM Kim NH, et al. Chronic thromboembolic pulmonary hypertension. Am J Respir Crit Care Med 2011; 183: 1605–1613. 21330453 10.1164/rccm.201011-1854CI

[4] Ende-Verhaar YM Cannegieter SC Vonk Noordegraaf A, et al. Incidence of chronic thromboembolic pulmonary hypertension after acute pulmonary embolism: a contemporary view of the published literature. Eur Respir J 2017; 49: 1601792. 28232411 10.1183/13993003.01792-2016

[5] Klok FA van der Hulle T den Exter PL, et al. The post-PE syndrome: a new concept for chronic complications of pulmonary embolism. Blood Rev 2014; 28: 221–226. 25168205 10.1016/j.blre.2014.07.003

[6] Sista AK Klok FA. Late outcomes of pulmonary embolism: the post-PE syndrome. Thromb Res 2018; 164: 157–162. 28641836 10.1016/j.thromres.2017.06.017

[7] Konstantinides SV Meyer G Becattini C, et al. 2019 ESC Guidelines for the diagnosis and management of acute pulmonary embolism developed in collaboration with the European Respiratory Society (ERS): The Task Force for the diagnosis and management of acute pulmonary embolism of the European Society of Cardiology (ESC). Eur Heart J 2019; 41: 543–603. 10.1093/eurheartj/ehz405 31504429

[8] Klok FA Tijmensen JE Haeck MLA, et al. Persistent dyspnea complaints at long-term follow-up after an episode of acute pulmonary embolism: results of a questionnaire. Eur J Intern Med 2008; 19: 625–629. 19046730 10.1016/j.ejim.2008.02.006

[9] Sanchez O Helley D Couchon S, et al. Perfusion defects after pulmonary embolism: risk factors and clinical significance. J Thromb Haemost 2010; 8: 1248–1255. 20236393 10.1111/j.1538-7836.2010.03844.x

[10] Klok FA van Kralingen KW van Dijk APJ, et al. Prevalence and potential determinants of exertional dyspnea after acute pulmonary embolism. Respir Med 2010; 104: 1744–1749. 20599368 10.1016/j.rmed.2010.06.006

[11] Stevinson BG Hernandez-Nino J Rose G, et al. Echocardiographic and functional cardiopulmonary problems 6 months after first-time pulmonary embolism in previously healthy patients. Eur Heart J 2007; 28: 2517–2524. 17670755 10.1093/eurheartj/ehm295

[12] Konstantinides SV Vicaut E Danays T, et al. Impact of thrombolytic therapy on the long-term outcome of intermediate-risk pulmonary embolism. J Am Coll Cardiol 2017; 69: 1536–1544. 28335835 10.1016/j.jacc.2016.12.039

[13] Kline JA Steuerwald MT Marchick MR, et al. Prospective evaluation of right ventricular function and functional status 6 months after acute submassive pulmonary embolism: frequency of persistent or subsequent elevation in estimated pulmonary artery pressure. Chest 2009; 136: 1202–1210. 19542256 10.1378/chest.08-2988 PMC2818852

[14] Nijkeuter M Hovens MMC Davidson BL, et al. Resolution of thromboemboli in patients with acute pulmonary embolism: a systematic review. Chest 2006; 129: 192–197. 16424432 10.1378/chest.129.1.192

[15] Alonso-Martínez JL Anniccherico-Sánchez FJ Urbieta-Echezarreta MA, et al. Residual pulmonary thromboemboli after acute pulmonary embolism. Eur J Intern Med 2012; 23: 379–383. 22560390 10.1016/j.ejim.2011.08.018

[16] Cosmi B Nijkeuter M Valentino M, et al. Residual emboli on lung perfusion scan or multidetector computed tomography after a first episode of acute pulmonary embolism. Intern Emerg Med 2011; 6: 521–528. 21461909 10.1007/s11739-011-0577-8

[17] Golpe R Llano LAPd Castro-Añón O, et al. Long-term outcome of patients with persistent vascular obstruction on computed tomography pulmonary angiography 6 months after acute pulmonary embolism. Acta Radiol 2012; 53: 728–731. 22850574 10.1258/ar.2012.110697

[18] Korkmaz A Ozlu T Ozsu S, et al. Long-term outcomes in acute pulmonary thromboembolism: the incidence of chronic thromboembolic pulmonary hypertension and associated risk factors. Clin Appl Thromb/Hemost 2012; 18: 281–288. 10.1177/1076029611431956 22275389

[19] Ribeiro A Lindmarker P Johnsson H, et al. Pulmonary embolism: one-year follow-up with echocardiography Doppler and five-year survival analysis. Circulation 1999; 99: 1325–1330. 10077516 10.1161/01.cir.99.10.1325

[20] Ludvigsson JF Andersson E Ekbom A, et al. External review and validation of the Swedish national inpatient register. BMC Public Health 2011; 11: 450–450. 21658213 10.1186/1471-2458-11-450 PMC3142234

[21] Öhman L Johansson M Jansson J-H, et al. Positive predictive value and misclassification of diagnosis of pulmonary embolism and deep vein thrombosis in Swedish patient registries. Clin Epidemiol 2018; 10: 1215–1221. 30271217 10.2147/CLEP.S177058 PMC6147534

[22] Eriksson M Holmgren L Janlert U, et al. Large improvements in major cardiovascular risk factors in the population of northern Sweden: the MONICA study 1986–2009. J Intern Med 2011; 269: 219–231. 21158982 10.1111/j.1365-2796.2010.02312.x

[23] Ho D, Imai K, King G, et al. MatchIt: nonparametric preprocessing for parametric causal inference. *J Stat Softw* 2011; 42: 1–28.

[24] Kahn SR Akaberi A Granton JT, et al. Quality of life, dyspnea, and functional exercise capacity following a first episode of pulmonary embolism: results of the elope cohort study. Am J Med 2017; 130: 990.e9–990.e21. 10.1016/j.amjmed.2017.03.033 28400247

[25] Lang IM Pesavento R Bonderman D, et al. Risk factors and basic mechanisms of chronic thromboembolic pulmonary hypertension: a current understanding. Eur Respir J 2013; 41: 462–468. 22700839 10.1183/09031936.00049312

