# Supplementary material for: Dyspnea after pulmonary embolism: a nation-wide population-based case–control study
Source: Pulm Circ. 2021 Sep 30;11(4):20458940211046831. doi: 10.1177/20458940211046831 (PMC8488523; doi:10.1177/20458940211046831)
Supplement: sj-pdf-1-pul-10.1177_20458940211046831 - Supplemental material for Dyspnea after pulmonary embolism: a nation-wide population-based case–control study [file sj-pdf-1-pul-10.1177_20458940211046831.pdf]

Dyspnea after pulmonary embolism: a nation-wide population-based case-control study

Lars T Nilsson, MD[1]; Therese Andersson, MD[1]; Flemming Larsen MD, PhD[2]; Irene M Lang, MD, PhD[3]; Per Liv, PhD[4]; Stefan Söderberg, MD, PhD[1]

[1]Department of Public Health and Clinical Medicine, Unit of Medicine, Umeå University, Umeå, Sweden

[2]Department of Molecular Medicine and Surgery, Section of Clinical Physiology, Karolinska Institute and Department of Clinical Physiology, Karolinska University Hospital, Stockholm, Sweden

[3]Department of Internal Medicine II, Division of Cardiology, Vienna General Hospital, Medical University of Vienna, Vienna, Austria

[4]Department of Public Health and Clinical Medicine, Section of Sustainable Health, Umeå University, Umeå, Sweden

Supplementary material

Content:

Supplementary Tables 1–8

Supplementary Tables 1-8

Table 1: Characteristics of patients in the post-PE group, comparisons between questionnaire responders and non-responders

Table 2: Characteristics of patients in the post-PE group, comparisons between patients included in regression models and those excluded because of young age and old age, respectively

Table 3: Univariable analysis of associations between covariates and exertional dyspnea

Table 4: Univariable analysis of associations between covariates and wake-up dyspnea

Table 5: Fully adjusted risk of exertional dyspnea in the post-PE group compared to the MONICA control group, stratified by age

Table 6: Fully adjusted risk of wake-up dyspnea in the post-PE group compared to the control group, stratified by age

Table 7. Comparison of self-reported health conditions between the sexes, including the direction of change, time to change, and degree of dyspnea, at 3.4 years after acute PE

Table 8. Self-reported comorbidities and their association with exertional dyspnea in the post-PE group

Supplementary Table 1. Characteristics of patients in the post-PE group, comparisons between questionnaire responders and non-responders

**Characteristic**	**Responders N=2105**	**Non-responders N=1405**	***P***
**Age at survey, mean (SD)**	65.4 (14.8)	70.1 (17.0)	<0.001
**Female sex**	1015 (48.2)	781 (55.6)	<0.001
**Pre-survey diagnoses, according to NPR**			
Congestive heart failure	143 (6.8)	228 (16.2)	<0.001
Cancer, any	222 (10.5)	186 (13.2)	0.015
Ischemic heart disease	291 (13.8)	320 (22.8)	<0.001
Cerebrovascular disease	116 (5.5)	179 (12.7)	<0.001
Atrial fibrillation	234 (11.1)	225 (16.0)	<0.001
Pulmonary embolism before 2005	231 (11.0)	205 (14.6)	<0.001

Data shown are numbers (%) of patients, unless otherwise indicated. PE: pulmonary embolism; NPR: Swedish National Patient Registry. *P*-values were based on Students t test or Pearson $\chi 2$ test.

Supplementary Table 2. Characteristics of patients in the post-PE group, comparisons between patients included in regression models and those excluded because of young age and old age, respectively

**Characteristic**	**Age <25 years N=36**	**Age 25–75 years N=1524**	**Age >75 years N=545**	***P***
**Female sex**	23 (63.9)	677 (44.4)	315 (57.8)	<0.001
**Pre-survey diagnoses, according to NPR**				
Congestive heart failure	0 (0.0)	66 (4.3)	77 (14.1)	<0.001
Cancer, any	2 (6.5)	161 (10.5)	59 (10.8)	0.742
Ischemic heart disease	0 (0.0)	149 (9.7)	142 (26.1)	<0.001
Cerebrovascular disease	0 (0.0)	73 (4.8)	43 (7.9)	0.009
Atrial fibrillation	0 (0.0)	128 (8.4)	106 (19.4)	<0.001
PE before 2005	0 (0.0)	140 (9.2)	91 (16.7)	<0.001
**Self-reported symptoms/diagnoses**				
Exertional dyspnea	14 (45.2)	774 (53.0)	320 (63.6)	<0.001
Wake-up dyspnea	3 (9.7)	181 (12.0)	66 (12.4)	0.892
Angina CCS 2	5 (16.7)	264 (17.8)	136 (26.5)	<0.001
Angina CCS 3-4	4 (12.9)	117 (8.0)	50(9.9)	0.279
Chest pain >30 min	5 (16.1)	238 (16.1)	98(19.4)	0.248
MI, self-reported	0 (0.0)	133 (9.2)	88 (17.3)	<0.001
Suspected MI, self-reported	0 ()	46 (3.2)	37 (7.3)	<0.001

PCI/CABG	1 (3.2)	108 (7.2)	46 (8.6)	0.375
Heart valve surgery	0 (0.0)	16 (1.1)	6 (1.1)	0.838
Hypertension	6 (20.0)	728 (48.5)	316 (59.2)	<0.001
COPD	1 (3.2)	128 (8.5)	40 (7.5)	0.472
Present smoking	8 (26.7)	139 (9.2)	17 (3.2)	<0.001
Previous smoking	8 (25.8)	754 (50.1)	190 (35.4)	<0.001

Data shown are numbers (%) of patients, unless otherwise indicated. The denominator for each ratio (%) is the number of valid responses for each item. PE: pulmonary embolism; CCS: Canadian Cardiovascular Society; MI: myocardial infarction; PCI: percutaneous coronary intervention; CABG: coronary artery bypass graft surgery; COPD: chronic obstructive pulmonary disease; NPR: Swedish National Patient Registry NPR: Swedish National Patient Registry. *P*-values were based on Pearson $\chi 2$ test.

Supplementary Table 3. Univariable analysis of associations between covariates and exertional dyspnea

Covariate	N	OR	95% CI
Study cohort (Post-PE/MONICA)	1461/1859	5.40	4.61–6.32
Sex (Women/Men)	1589/1731	1.52	1.32–1.76
Age (1-year age groups)	3320	1.04	1.04–1.05
COPD (Yes/No)	137/3170	14.09	8.63–23.00
Heart valve surgery (Yes/No)	24/3286	2.42	1.08–5.43
Hypertension (Yes/No)	1217/2082	2.70	2.32–3.14
Present smoking (Yes/No)	455/2841	1.00	0.81–1.23
Previous smoking (Yes/No)	1325/1679	1.54	1.32–1.79
Congestive heart failure (Yes/No)	65/3255	6.47	3.66–11.43
Cancer (Yes/No)	292/3028	1.23	0.96–1.58
Ischemic heart disease (Yes/No)	187/3133	4.06	2.08–5.54
Cerebrovascular disease (Yes/No)	80/3240	2.18	1.40–3.40
Atrial fibrillation (Yes/No)	141/3179	3.06	2.17–4.32

Data shown are odds ratios (OR) with 95% confidence intervals (CI). The odds that exertional dyspnea was related to the indicated comorbidities were tested with univariable logistic regression analysis, based on both the post-PE and MONICA control groups. PE: pulmonary embolism; MONICA: multinational MONItoring of trends and determinants in CArdiovascular disease health survey; COPD: chronic obstructive pulmonary disease

Supplementary Table 4. Univariable analysis of associations between covariates and wake-up dyspnea

Covariate	N	OR	95% CI
Study cohort (Post-PE/MONICA)	1506/1893	7.70	5.28–11.23
Sex (Women/Men)	1638/1761	1.40	1.06–1.85
Age (1-year age groups)	3399	1.04	1.03–1.05
COPD (Yes/No)	144/3243	8.53	5.80–12.55
Heart valve surgery (Yes/No)	24/3370	4.00	1.48–10.82
Hypertension (Yes/No)	1252/2125	2.66	2.00–3.54
Present smoking (Yes/No)	464/2913	0.93	0.61–1.40
Previous smoking (Yes/No)	1360/1719	1.23	0.93–1.64
Congestive heart failure (Yes/No)	71/3328	5.00	2.85–8.79
Cancer (Yes/No)	301/3098	1.00	0.62–1.63
Ischemic heart disease (Yes/No)	193/3206	4.06	2.75–5.99
Cerebrovascular disease (Yes/No)	88/3311	3.49	2.00–6.12
Atrial fibrillation (Yes/No)	148/3251	3.85	2.48–5.95

Data shown are odds ratios (OR) with 95% confidence intervals (CI). The odds that wake-up dyspnea was related to the indicated comorbidities were tested with univariable logistic regression analysis, based on both the post-PE and MONICA control groups. PE: pulmonary embolism; MONICA: multinational MONItoring of trends and determinants in CArdiovascular disease health survey; COPD: chronic obstructive pulmonary disease

Supplementary Table 5. Fully adjusted risk of exertional dyspnea in the post-PE group compared to the MONICA control group, stratified by age

Age, years	OR	95% CI	N (post-PE/MONICA)	Exertional dyspnea, N
25–34.9	7.94	4.12–15.30	65/306	51
35–44.9	7.31	4.33–12.34	124/354	98
45–54.9	4.96	3.18–7.74	184/372	133
55–64.9	3.66	2.64–5.08	464/406	328
65–75.9	2.97	2.24–3.94	599/412	464

Data shown are odds ratios (OR) with 95% confidence intervals (CI), which indicated the odds that exertional dyspnea was related to the study cohort (post-PE vs. MONICA control group), in each age group, after adjusting for all significant covariates (chronic obstructive pulmonary disease, ischemic heart disease, congestive heart failure, hypertension, sex). PE: pulmonary embolism; MONICA: multinational MONItoring of trends and determinants in CArdiovascular disease health survey

Supplementary Table 6. Fully adjusted risk of wake-up dyspnea in the post-PE group compared to the control group, stratified by age

Age, years	OR	95% CI	N (post-PE/MONICA)	Wake up dyspnea, N
25–34.9	28.54	3.35–243.21	66/309	7
35–44.9	15.39	4.24–57.77	125/359	21
45–54.9	17.97	4.03–80.08	189/379	22
55–64.9	2.40	1.27–4.51	475/418	63
65–75.9	3.50	1.84–6.62	624/420	93

Data shown are odds ratios (OR) with 95% confidence intervals (CI), which indicated the odds that wake-up dyspnea was related to the study cohort (post-PE vs. MONICA control group), in each age group, after adjusting for all significant covariates (chronic obstructive pulmonary disease, ischemic heart disease, atrial fibrillation, hypertension, sex). PE: pulmonary embolism; MONICA: multinational MONItoring of trends and determinants in CArdiovascular disease health survey

Supplementary Table 7. Comparison of self-reported health conditions between the sexes, including the direction of change, time to change, and degree of dyspnea, at 3.4 years after acute PE

Condition	Men, N (%)	Women, N (%)	*P*
**Change in health after PE**			<0.001
Improved	534 (49.4)	400 (39.9)	
Unchanged	334 (30.9)	355 (35.4)	
Deteriorated	212 (19.6)	247 (24.7)	
**Time to improvement**			0.011
Immediately	259 (50.6)	150 (40.3)	
Within 6 months	211 (41.2)	182 (48.9)	
Within 2 years	32 (6.3)	25 (6.7)	
After 2 years	10 (2.0)	15 (4.0)	
**Time to deterioration**			0.365
Immediately	95 (48.2)	114 (50.4)	
Within 6 months	47 (23.9)	46 (20.4)	
Within 2 years	29 (14.7)	44 (19.5)	
After 2 years	26 (13.2)	22 (9.7)	
**Dyspnea upon exertion**			<0.001
No	358 (34.5)	224 (23.1)	
Two stairs	413 (39.7)	386 (39.9)	
One stair	220 (21.2)	291 (30.1)	
At rest	48 (4.6)	67 (6.9)	

Data shown are numbers (%) of patients. The denominator for each ratio (%) is the number of valid responses for each item. Differences between men and women were evaluated with the Pearson $\chi 2$ test; PE: pulmonary embolism.

Supplementary Table 8. Self-reported comorbidities and their association with exertional dyspnea in the post-PE group

Comorbidity	N (%)	OR (95% CI)
Chronic disease, any	1233 (61.4)	2.74 (2.27–3.31)
Cancer	159 (7.9)	1.23 (0.88–1.72)
Hematologic disease	52 (2.6)	2.01 (1.10–3.66)
Intravenous line, long term	22 (1.1)	5.02 (1.47–17.19)
Peripheral arterial disease	6 (0.3)	–
Cerebrovascular disease	165 (7.5)	1.19 (0.84–1.69)
Splenectomy	19 (0.9)	1.02 (0.37–2.77)
Pulmonary disease	123 (6.1)	4.02 (2.48–6.52)
Heart disease	291 (14.5)	2.53 (1.88–3.39)
Pacemaker/ICD	61 (3.0)	2.05 (1.13–3.70)
Ventricular shunt	6 (0.3)	2.31 (0.40–13.42)
Inflammatory bowel disease	43 (2.1)	2.24 (1.13–4.44)
Chronic inflammatory disease	88 (4.4)	2.30 (1.39–3.79)
Previous trauma	110 (5.5)	1.24 (0.83–1.87)
Other chronic disease	380 (18.9)	2.06 (1.61–2.64)

Data shown are numbers (%) of patients. The denominator for each ratio (%) is the number of valid responses for each item. The odds ratios (OR) with 95% confidence intervals (CI) indicate the risk (adjusted for age and sex) that exertional dyspnea was related to self-reported comorbidities. PE: pulmonary embolism; ICD: implantable cardioverter defibrillator
